# Gender affirmation among transgender and gender-diverse people in middle age and late life: a scoping review

**DOI:** 10.1093/geront/gnag194

**Published:** 2026-08-12

**Authors:** Chenxi Yang, Yu-Te Huang, Yue Xu, Peter A Newman

**Affiliations:** Department of Social Work and Social Administration, The University of Hong Kong, Hong Kong, Mainland China; Department of Social Work and Social Administration, The University of Hong Kong, Hong Kong, Mainland China; Department of Social Work and Social Administration, The University of Hong Kong, Hong Kong, Mainland China; Factor-Inwentash Faculty of Social Work, University of Toronto, Toronto, Ontario, Canada

**Keywords:** Transgender, Gender affirmation, Old age, Aging, Review

## Abstract

**Background and Objectives:**

Although gender affirmation is a social determinant of health for transgender and gender-diverse people, reviews have predominantly focused on the medical domain and on younger populations. We conducted a scoping review by mapping peer-reviewed studies on 4 domains of gender affirmation among transgender and gender-diverse people in middle age and late life.

**Research Design and Methods:**

Using the Joanna Briggs Institute’s scoping review methodology and PRISMA-ScR reporting guidelines, we conducted a literature search of the following databases: MEDLINE, Social Work Abstracts, CINAHL PLUS from EBSCOhost, APA PsycINFO, ASSIA, Sociological Abstracts from ProQuest, and SCOPUS. Of the 1,339 studies identified, 58 were included in this review. We used a hybrid thematic content analysis to synthesize the key findings.

**Results:**

The participants reported both gender-affirming and non-affirming experiences in (a) the psychological domain, which includes identity confusion, dysphoria, internalized stigma, and self-acceptance and identity realization in later life; (b) the social domain, which encapsulates decisions to conceal or disclose their gender identity, experiences of rejection or support, and community engagement and advocacy actions; (c) the medical domain, which concerns access to and experiences with gender-affirming care and non-affirming services in healthcare; and (d) the legal domain, which involves pursuing gender recognition, navigating legal documentation, and facing systemic barriers in end-of-life planning.

**Discussion and Implications:**

This review highlights the multi-dimensional needs associated with gender affirmation and the systemic barriers faced by transgender and gender-diverse adults in middle age and late life. It provides insight into gender-affirming experiences that can inform more age-friendly and gender-affirming care.

Transgender and gender-diverse people are those whose gender identity does not align with the culturally prescribed expectations associated with their assigned sex at birth. This population includes both people who identify within the gender binary system (e.g., transgender men, transgender women), those who identify outside of it (e.g., gender non-binary or nonconforming, genderqueer), and those who identify as “neither female nor male, as both, or as somewhere in between” (Beemyn, 2005, p. 1). For these individuals, gender affirmation—the psychological, social, medical, and legal validation of their gender identity—is of major importance in their lives (King & Gamarel, 2021; Lelutiu-Weinberger et al., 2020; Lorusso et al., 2025; Reisner et al., 2016). As an essential part of identity and sense of self, affirmation occurs when a transgender and gender-diverse person is recognized for who they truly are and treated as they wish to be treated (Sevelius, 2013). This affirmation leads to positive psychological, physical, and social changes and, consequently, enhances overall health and well-being (Almazan & Keuroghlian, 2021; Fowler et al., 2024; Hughto et al., 2020).

Gender affirmation includes four dimensions (Reisner et al., 2016). The psychological aspect encompasses self-acceptance and internal satisfaction with one’s gender identity and expression (Dorsen et al., 2022; Reisner et al., 2016; Sevelius, 2013). The social aspect involves disclosing one’s gender identity and experiences to others, surrounding oneself with people who support and validate this identity, and receiving support from family and peers (King & Gamarel, 2021). The medical aspect refers to hormones, surgery, and other procedures that contribute to an alignment between an individual’s physical appearance and their gender identity (Reisner et al., 2016). The legal aspect includes actions such as updating identity documents to ensure that they match a person’s preferred name and gender identity (Austin et al., 2022; Pamfile et al., 2025). However, due to structural barriers and the societal stigma associated with transphobia, individuals pursuing gender affirmation in various aspects may go through both gender-affirming and non-affirming experiences. Non-affirming experiences can include gender dysphoria and internalized transphobia at the psychological level; gender-based rejection from family and social groups at the social level; the inaccessibility of gender-affirming care at the medical level; and the impossibility of obtaining gender recognition at the legal level.

Although the literature explores the gender-affirming experiences of transgender and gender-diverse people in these four domains, reviews have focused more on the medical aspect (Kearns et al., 2024; Mills et al., 2022; Surendran et al., 2025; Swan et al., 2023; White Hughto & Reisner, 2016). Recent studies have expanded to examine how social and legal dimensions shape transgender health, although these are exclusively quantitative studies (King & Gamarel, 2021; Scheim et al., 2025). Even the few reviews of research focusing on middle-aged and older adults resolve around medical affirmation (Asti et al., 2026; Mehta et al., 2024), leaving psychological, social, and legal experiences understudied. Although these reviews have valuable implications for transgender health, they offer limited discussions of gender affirmation for middle-aged and older adults.

Transgender and gender-diverse middle-aged and older adults constitute a small but important segment of the population, estimated at 0.42% of U.S. adults aged 35 to 64 and 0.26% of those 65 and older (Herman & Flores, 2025). Compared with their younger counterparts, this group faces unique needs and experiences in gender affirmation, such as identity development, psychological adjustment to aging, and access to social, medical, and legal support and resources (Fredriksen-Goldsen & Muraco, 2010). Although these individuals represent diverse birth cohorts with different social backgrounds, many have spent a significant portion of their lives in environments characterized by ambivalent or negative social attitudes toward transgender and gender-diverse people (Nuttbrock et al., 2009), by a near absence of resources for medical and legal affirmation (Barsigian et al., 2020; Carvalho et al., 2025; Jesperson & Wesley, 2023; Siverskog, 2014), and by the institutional pathologization of their identities. For example, it was only in 2013 that the American Psychiatric Association replaced “gender identity disorder” with “gender dysphoria” in the DSM-5 (American Psychiatric Association, 2013), and in 2018 that the World Health Organization removed “transsexuality” from the “Mental and Behavioral Disorders” chapter (World Health Organization, 2019). With the growing socio-cultural acceptance and increased attention to the need for gender-affirming resources, middle age and late life present both opportunities and challenges related to gender affirmation, psychologically, socially, medically, and legally, for transgender and gender-diverse adults. This group includes those who recognized their gender identity early in life but went through largely non-affirming periods, and those who have recently embraced this identity and/or are contemplating gender affirmation in middle age and beyond.

## The current study

This review aims, first, to move beyond the mainly medical focus of prior reviews by including psychological, social, and legal domains, as well as qualitative studies and, second, to specifically explore gender affirmation among transgender and gender-diverse people aged 40 and older. Although the age of 40 is generally considered young for aging research, we selected this cutoff (Hsieh, 2014; Thomas et al., 2023; Wang et al., 2021) to recognize the phenomenon of accelerated aging within the transgender and gender-diverse community (Epel, 2020; Madhav et al., 2025). For this community, multiple forms of marginalization and stress often lead to an early onset of frailty and poor health (Kneale et al., 2024). This threshold also mirrors established government frameworks, such as Indonesian policies that classify people aged 40 and older as part of the aging demographic (Wolter et al., 2025). Our work focuses on experiences of gender affirmation within this community, from early identity affirmation (or non-affirmation) to the impact of these experiences in middle age and late life. We drew on the four dimensions of gender affirmation identified by Reisner et al. (2016), namely the psychological, social, medical, and legal dimensions, as a conceptual framework to guide the synthesis. While previous reviews focusing on middle-aged and older adults have concentrated exclusively on the medical domain (Asti et al., 2026; Mehta et al., 2024), our review is among the first to map gender affirmation by jointly examining these four domains, specifically for this population.

## Method

We conducted a scoping review to provide an overview of research findings regarding gender affirmation for transgender and gender-diverse people in middle age and late life (Peters et al., 2020; Tricco et al., 2018). We included both transgender and gender-diverse people who had recognized their gender identity early in life and those whose identity was newly emerging in middle age and late life. This scoping review answered the following question: What are the types, characteristics, and distribution of the literature on the gender-affirming (and non-affirming) experiences of transgender and gender-diverse people in middle age and late life in the psychological, social, medical, and legal domains? Scoping reviews are better suited than other types of reviews (e.g., systematic reviews) to answer exploratory research questions and to map key concepts, evidence, and gaps underpinning a field of research (Arksey & O’Malley, 2005; Heyn et al., 2019). We followed the Joanna Briggs Institute guidelines (2015) and the framework developed by Arksey and O’Malley (2005) to conduct our scoping review. The main steps were as follows: (1) identify the research questions; (2) define a search strategy; (3) develop the inclusion and exclusion criteria; (4) map the data; and (5) collate, summarize, and report the results. The review protocol was registered on the Open Science Framework (OSF) (https://osf.io/q8g3r).

### Information sources and search strategy

Following previous reviews of transgender and LGBTQ+ older adults (Perone et al., 2025; Yang et al., 2025), we conducted a literature search in April 2025 in the following databases: MEDLINE, Social Work Abstracts, CINAHL PLUS from EBSCOhost, APA PsycInfo, ASSIA, Sociological Abstracts from ProQuest, and SCOPUS. The search terms included those associated with transgender and gender-diverse people, gender affirmation, and middle-aged and older adults (see Supplementary Appendix 1). These terms were adapted from published systematic reviews (Asti et al., 2026; Catlett, 2024; Fredriksen Goldsen et al., 2019; King & Gamarel, 2021; Yang et al., 2025) and refined by the research team to capture gender-affirming experiences in psychological, medical, social, and legal settings. A manual search of the reference lists of all relevant reviews and identified studies, as well as a search on Google Scholar, were conducted in June 2025 to identify additional relevant studies (see Supplementary Appendix 2).

### Study selection criteria

Studies were selected based on the following inclusion and exclusion criteria. The selected publications had to (a) be peer-reviewed, original research published in English; (b) include transgender and gender-diverse participants aged 40 and older; (c) include data on experiences of gender affirmation (or non-affirmation), encompassing psychological, social, medical, and legal aspects; and (d) have been published after January 1, 2000. We started our study period in 2000 because transgender and gender-diverse older people were invisible until around that year (Witten, 2016). If a study did not explicitly mention whether the participants had reached middle age or late life, we chose an age cutoff of 40 years to identify them. The data on gender-affirming (or non-affirming) experiences included those explicitly described as such by the authors in the source study, and those interpreted by the review team as experiences of affirmation (or non-affirmation) based on established definitions of gender affirmation developed by Reisner et al. (2016) and Sevelius (2013).

Studies were excluded if they (a) did not report primary data (e.g., literature reviews, descriptive articles, conference abstracts, protocols, nonempirical studies); (b) did not include data specific to gender-affirming experiences; or (c) did not include data specific to transgender and gender-diverse people in middle age and late life (i.e., aged ≥ 40).

### Study selection process

The search results were collated using the Covidence systematic review software (Veritas Health Innovation, Melbourne, Australia), and duplicates were removed. During the initial screening step, two authors (C.Y., Y.X.) independently evaluated the titles and abstracts for inclusion. We organized team discussions to ensure consistent application of the inclusion and exclusion criteria, resulting in agreement in 90% of the cases (Cohen’s kappa = 0.79, indicating substantial agreement) (Landis & Koch, 1977). The same reviewers then independently examined the full-text articles for inclusion. All discrepancies were resolved during discussions between these reviewers.

### Data extraction and synthesis

Two authors (C.Y., Y.X.) extracted the following data for analysis: publication characteristics (i.e., author(s), year, country/location), methods (i.e., qualitative, quantitative, mixed methods), sample size, sample characteristics, funding sources, and key findings relevant to gender affirmation.

The publications were then categorized by domain of gender affirmation. We synthesized the key findings using an inductive/deductive hybrid thematic analysis, a process combining deductive coding (derived from the conceptual framework, i.e., the four domains of gender affirmation) and inductive coding (themes emerging from the data) (Fereday & Muir-Cochrane, 2006). This approach allowed us to systematically address gender affirmation in the four domains established by Reisner et al. (2016) while enriching our analysis through the narratives of the participants. In the first stage, we deductively used the a priori framework to categorize the data into the four dimensions of gender affirmation (i.e., psychological, social, medical, and legal). In the second stage, we applied an inductive approach within these four themes, creating codes through a line-by-line reading of the data to capture their meanings. Subthemes were then generated to address the research question and capture the meanings shared by groups of initial codes. In the final stage, we re-examined the data and refined the subthemes.

When the studies covered broad age ranges (e.g., Balcerek et al., 2021; Cai et al., 2019), we only extracted data if the authors separated their findings by age, excluding those that grouped our target demographic (aged 40 and older) with younger populations (aged 39 and younger). To integrate the quantitative findings with subjective experiences, all variables associated with gender affirmation (e.g., quality-of-life scores) were conceptualized as the implications of gender-affirming or non-affirming experiences. Data from the Results sections of quantitative studies or the quantitative components of mixed-methods studies were qualitatively coded. For example, statistical data demonstrating an association between gender-affirming medical treatment and higher quality-of-life scores (Cai et al., 2019, pp. 36–37) were coded as a positive correlate of medical affirmation and integrated into the subtheme “Gender-affirming hormones and surgeries.” The results are reported in accordance with the PRISMA-ScR guidelines (Tricco et al., 2018).

## Results

**Figure 1:**
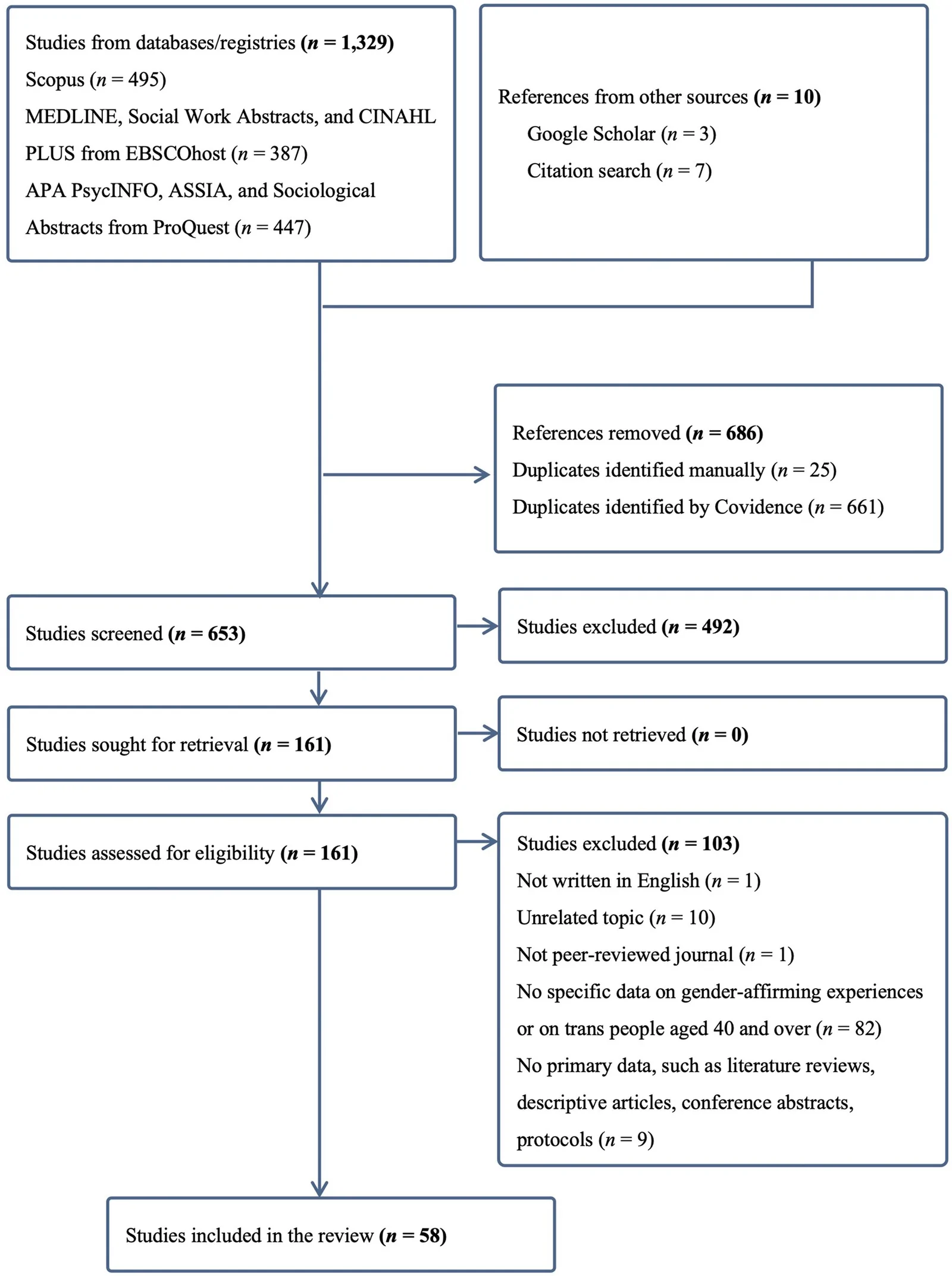
PRISMA flow diagram of the literature search process.

**Figure 2:**
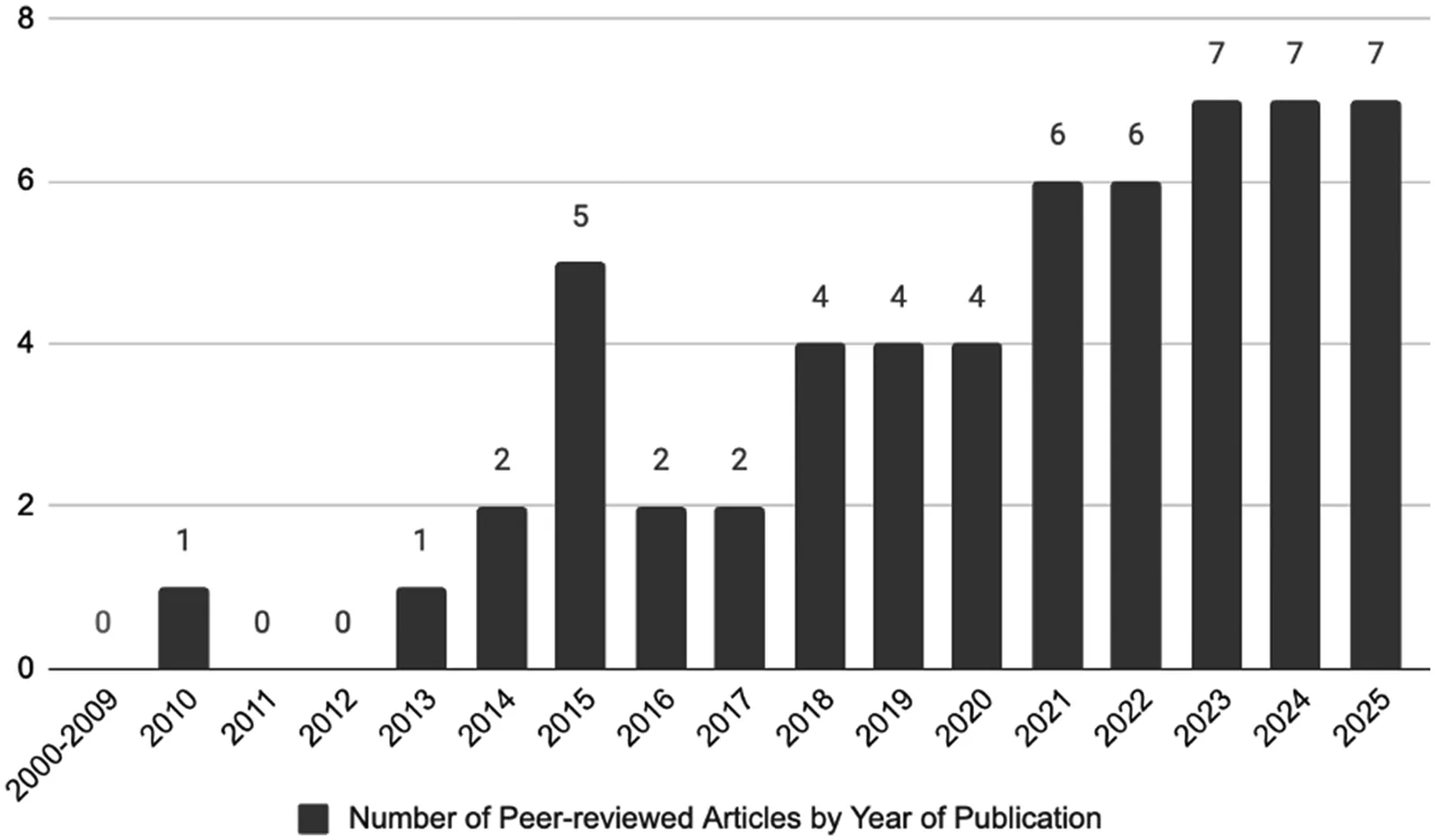
Distribution of peer-reviewed articles by year of publication (*n* = 58).

The database search identified 653 nonduplicate titles and abstracts; of these, 161 full-text articles were assessed against the inclusion/exclusion criteria, and 58 were ultimately included in this review (Figure 1). Supplementary Appendix 3 presents the characteristics of the 58 included studies published between 2010 and 2025, showing that this body of literature has grown considerably over the past decade (Figure 2). No studies were published between 2000 and 2009. Only four studies were published between 2010 and 2014. In contrast, nearly half of the included studies (*n *= 27) were published after 2022. Overall, 67% of the studies (*n *= 39) reported funding sources, 10% (*n *= 6) reported receiving no funding, and 22% (*n *= 13) did not mention any funding sources. Among the studies that reported funding sources, the majority (77%, *n *= 30) were funded by government or academic institutions, and 33% (*n *= 13) received funding from organizations. However, we did not observe any meaningful patterns regarding funding sources among the included studies.

The studies were predominantly conducted in high-income countries: the largest number of studies were conducted in the United States (*n *= 27), followed by Canada (*n *= 7), the United Kingdom (*n *= 5), Sweden (*n *= 3), South Africa, Australia, and India (*n *= 2 each), and Thailand, New Zealand, Portugal, Ireland, Chile, Argentina, and Indonesia (*n *= 1 each). Two studies were conducted in 24 countries (e.g., the United States, Canada, Thailand), and another was conducted in Portugal and Spain. Between 2010 and 2016, research was predominantly conducted in the United States, with a slight expansion into the Global North. From 2017 onward, research began to emerge in Chile, South Africa, Argentina, Indonesia, India, and Thailand, although the literature from the Global North continued to dominate the field. Nearly half of the included studies (*n *= 28) were conducted by 11 recurring research groups, mainly based in the Global North (*n *= 26), including six American research teams that produced 18 studies. Furthermore, 21 studies relied on nine shared datasets. Specifically, 19 studies used eight datasets from the Global North, including 11 studies that relied on four datasets predominantly from the United States. This suggests that the evidence base is disproportionately shaped by a narrow range of perspectives from researchers and study populations. Most of the studies (82.8%, *n *= 48) were qualitative, 8.6% (*n *= 5) were quantitative, and 8.6% (*n *= 5) used mixed methods. Twenty-five (43.1%) studies discussed the psychological aspect of gender-affirming experiences, 40 (69.0%) discussed the social aspect, 39 (67.2%) discussed the medical aspect, and 10 (17.2%) discussed the legal aspect.

Of the 58 studies, 33 focused exclusively on transgender and gender-diverse people in middle age and late life. The remaining studies examined LGBTQ+ or transgender and gender-diverse populations of different ages; for the purpose of this review, disaggregated data regarding transgender and gender-diverse people aged 40 and older were extracted. In total, 1,838 participants were represented in this scoping review. Among the studies that reported gender identities, transgender women were predominant, followed by transgender men. Since 2020, the literature has shown a modest but growing representation of genderqueer, gender non-conforming, gender-fluid, and non-binary individuals. Notably, many studies used the umbrella term “transgender” without specifying gender subgroups. The literature primarily focused on participants aged 50, 60, and older. In 26 studies reporting race/ethnicity, the transgender and gender-diverse samples predominantly identified as White. Only seven studies provided disaggregated data on the participants’ socioeconomic status, with several noting that most of the participants came from middle or low socioeconomic backgrounds, but had completed university studies.

Having presented the descriptive characteristics of the included studies, the following paragraphs detail the thematic findings. To clearly present the participants’ experiences of gender affirmation and non-affirmation, we organized the themes into four domains, namely psychological, social, medical, and legal, each detailed below alongside its subthemes. The key themes and subthemes are presented in Table 1.

**Table 1:** Themes, subthemes, and studies associated with each subcategory.

Themes	Subthemes	Studies (*n*)
Psychological aspect	Gender identity challenges	16
	Impact of aging on psychological gender affirmation	4
	Self-acceptance and self-actualization	15
Social aspect	Concealing or revealing transgender and gender-diverse identities	14
	Social relationships and interactions	34
	Community building and advocacy	8
Medical aspect	Gender-affirming hormones and surgeries	21
	Gender-affirming and non-affirming health services	27
Legal aspect	Navigating legal and institutional recognition of gender identity	8
	Gender-affirming strategies in end-of-life documents	4

### Psychological domain

Three subthemes were identified within the psychological domain. The first subtheme, “Gender identity challenges,” highlights how the participants have experienced gender identity confusion, internalized stigma, dysphoria, and resilience throughout their lives. The second subtheme, “Impact of aging on psychological gender affirmation,” explores how physical changes and health problems, such as menopause and cognitive decline, influence the affirmation of their identity. The final subtheme, “Self-acceptance and self-actualization,” describes how transgender and gender-diverse individuals experience acceptance of their gender identity in middle age and late life.

#### Gender identity challenges

Eight studies mentioned the participants’ early experiences of identity exploration or awareness during childhood, adolescence, or young adulthood. During this period, the participants often felt confused about their gender identity and expressed feelings of “ambiguity” (Carvalho et al., 2024; Fabbre, 2017; Walker et al., 2016). One participant recalled becoming aware of their gender at the age of 3 or 4 and described an attraction to wearing feminine clothing (Siverskog, 2014), while another recounted having prayed, since childhood, to wake up as a girl (Jesperson & Wesley, 2023). However, this period of their lives was marked by a lack of social acceptance of gender exploration and affirmation (Jesperson & Wesley, 2023; Siverskog, 2014), as well as limited access to the information and language needed to describe their transgender and gender-diverse identity (Barsigian et al., 2020; Willis et al., 2021). Only two articles discussed cases of early affirmation, where the participants found terms like “transvestite” or “two-spirit” that resonated with them in late adolescence or young adulthood (Jesperson & Wesley, 2023, p. 275; Willis et al., 2021, p. 2801).

Both transgender and gender-diverse people who recognized their identity early in life and those whose identity emerged in middle age and late life experienced either initial or persistent challenges related to their gender identity in later life. Twelve articles highlighted the participants’ experiences of discomfort and stigma related to gender identity in middle age and late life. These experiences included feelings of gender dysphoria (Banerjee & Rao, 2020; Cook-Daniels & Munson, 2010; Hurd & Mahal, 2025), internalized stigma (Baril & Silverman, 2024; Fabbre & Gaveras, 2020; Siverskog, 2014), and self-rejection or suppression of gender identity (Fabbre, 2015, 2017). Some of the participants described their gender as their “deep dark secret” (Fabbre & Gaveras, 2020, p. 353), and many feared that others would discover this secret (Baril & Silverman, 2024; Siverskog, 2014). These struggles led some of the participants to feel that they had wasted their lives, were “failures” (Carvalho et al., 2025; Fabbre, 2014, p. 169; Willis et al., 2021), or were suffering from depression (Fabbre, 2015). However, other participants demonstrated resilience by resisting transphobia (Walker et al., 2016) or confronting discrimination through empowerment strategies (Fabbre & Gaveras, 2020; Reygan & Henderson, 2019).

#### Impact of aging on psychological gender affirmation

Four articles revealed the complex implications of aging and health problems on the participants’ psychological experiences of gender affirmation (Baril & Silverman, 2024; Siverskog, 2015; Xin & Lane, 2025). A trans man in Sweden reported feeling less discomfort with his aging body and gender expression, while a trans woman described positive experiences as her body became “more androgynous with age” (Siverskog, 2015, p. 12). However, one study highlighted the negative effect on a trans man’s mental well-being of his inability to practice binding (a gender-affirming practice) due to dementia (Baril & Silverman, 2024).

Two articles reported on menopause experiences that brought participants closer to, or further from, feeling like a woman, a man, or a non-binary person; in some cases, these experiences made them feel more grounded and secure in their identity (Toze & Westwood, 2025; Xin & Lane, 2025). For example, going through menopause helped a 55-year-old transmasculine person in Australia feel like they were “rid of anything that was female” (Xin & Lane, 2025, p. 292). Similarly, menopause helped a gender non-conforming person (aged 50–59) in the United Kingdom to feel that they were not “fully female” (Toze & Westwood, 2025, p. 450), which facilitated their gender affirmation.

#### Self-acceptance and self-actualization

Fifteen articles described the participants’ experiences of identity exploration, self-acceptance, and self-actualization of their gender identity in middle age and late life. In some studies, the participants expressed a desire to be transgender or female and found that the term “transgender” aligned with their experiences in middle age (Katz-Wise & Budge, 2015; Walker et al., 2016; Willis et al., 2020). A 65-year-old trans woman in the United States shared that a significant life event (e.g., marital difficulties) triggered an “epiphany” that made her realize that she “couldn’t deny who [she] was” (Fabbre, 2017, p. 484).

Some older adults described participation in certain activities, such as bondage, discipline, sadism, and masochism practices, as conducive to their psychological gender affirmation (Cook-Daniels & Munson, 2010). In three studies, the participants found that engaging in spiritual conversations helped them accept and/or cope with their gender identity (Banerjee & Rao, 2020; Bower et al., 2023; Fabbre et al., 2023). This acceptance was described as having a positive impact (Edwards et al., 2023; Gaveras et al., 2023; Jesperson & Wesley, 2023; Katz-Wise & Budge, 2015; Willis et al., 2021). A 53-year-old trans woman in the United States explained that the development of her transgender identity had brought her “tremendous joy and discovery” and had helped her find “a sense of true self” (Katz-Wise & Budge, 2015, p. 164). A 72-year-old trans man in the United States expressed that embracing his transgender identity had allowed him to “be able to live instead of exist” (Adan et al., 2021, p. 339). Notably, three articles reported that for transgender and gender-diverse people in middle age and late life, aging promoted acceptance of their gender identity (Carvalho et al., 2024; Fabbre, 2014; Willis et al., 2021). For those who had struggled for decades with their gender identity, the discovery of others who had transitioned in their late 50 s and the realization of their own aging left them with a feeling that they “don’t have much time left” (Fabbre, 2014, p. 167); this prompted them to live their lives to the fullest and to embrace their identity (Carvalho et al., 2024; Fabbre, 2014; Willis et al., 2021).

### Social domain

Three subthemes were identified within the social domain. The first subtheme, “Concealing or revealing transgender and gender-diverse identities,” highlights the challenges, motivations, and experiences related to navigating this visibility in various social settings. The second subtheme, “Social relationships and interactions,” explores the participants’ experiences of acceptance, validation, or rejection due to their gender identity within social networks, as well as the health consequences of social non-affirmation. The final subtheme, “Community building and advocacy,” describes the participants’ experiences of supporting and empowering their younger peers.

#### Concealing or revealing transgender and gender-diverse identities

Four studies discussed the participants’ concealment of their gender expression in early life, often driven by fear of being rejected by their parents and teachers (Fabbre & Gaveras, 2020; Jesperson & Wesley, 2023; Siverskog, 2014; Willis et al., 2021). However, deciding whether to reveal or conceal one’s gender identity in middle age and late life presented a unique and considerable challenge. As many participants had established their social roles as employees, spouses, or parents, revealing their gender identity (i.e., coming out) carried risks such as unemployment and rejection by their chosen family.

Twelve articles examined the participants’ desires and experiences related to concealing or revealing their gender identity to others in middle age and late life. Many participants chose to conceal their identity in public (Cashman, 2018; Kia et al., 2025), at work (Katz-Wise & Budge, 2015), or within their family (Fabbre, 2015; Fabbre & Gaveras, 2020). Some of the participants in India and the United States revealed having concealed their identity to conform to social and family expectations and roles (Fabbre, 2014, 2017; Katz-Wise & Budge, 2015; Thelly et al., 2025). For example, one trans woman concealed her identity at work to avoid losing her job, which would have compromised her ability to finance her daughter’s college education; however, she expressed a desire to transition once her daughter graduated (Katz-Wise & Budge, 2015). Similarly, another trans woman, married and a mother of two, explained that she concealed her identity at work, within a U.S. government agency: “I’ve got a house, and children. I am—I am the provider there. And if I lost my job, what am I gonna do? I love my children. So, what am I gonna do?” (Fabbre, 2017, p. 483). Although some trans women described periods in their past as times when they “did what they were supposed to do,” others felt that they had “served time” by conforming to social expectations or roles (Fabbre, 2014, p. 168).

Other people discussed their experiences of “passing,” a term used to describe being seen as cisgender and/or concealing one’s transgender and gender-diverse identity and history. One trans woman noted that as her voice was an obstacle to passing as a cis woman, she strategically remained silent in bathrooms (Cashman, 2018). Similarly, one trans man chose to “go back into the closet” later in life to maintain harmonious relationships with those around him (Siverskog, 2014, p. 393). However, some of the participants noted that passing compromised their ability to live authentically, while others described practical difficulties associated with passing (Hurd & Mahal, 2025; Knochel & Flunker, 2021). For instance, a 62-year-old Swedish trans man, who had come out to only a few people and whose identity as a man was otherwise unquestioned, described the constraints related to toilet use in his daily life: “I couldn’t stand up and pee as he does, so I don’t go at all” (Siverskog, 2015, p. 9).

Four studies detailed the participants’ motivations and experiences of revealing their transgender and gender-diverse identity to others (e.g., coming out). For example, a genderqueer person in Sweden expressed a desire not to be categorized based on binary gender norms (Siverskog, 2015), while many trans women in the United States wanted others to validate their identified gender (Katz-Wise & Budge, 2015). The participants shared their ways of coming out, including changing their gender expression through hairstyles, clothing, and names, in middle age and late life (Cashman, 2018; Jesperson & Wesley, 2023).

#### Social relationships and interactions

Thirty-one studies explored the participants’ social relationships and interactions in various contexts in middle age and late life, including in public spaces, workplaces, within family and circles of friends, religious communities, social groups, and the broader LGBTQ+ community. Multiple studies discussed the participants’ experiences of invisibility, harassment, and discrimination in public settings, particularly on the streets and when using public transportation (Hurd & Mahal, 2025; Miedema et al., 2022; Reygan et al., 2022), while age-related prejudices further ostracized transgender and gender-diverse older people (Banerjee & Rao, 2020). The participants also described their experiences and concerns about workplace discrimination in settings such as educational and government institutions (Adan et al., 2021; Cook-Daniels & Munson, 2010; Fabbre, 2015, 2017; Hurd & Mahal, 2025; Katz-Wise & Budge, 2015; Pang et al., 2019; Siverskog, 2014; Willis et al., 2021).

Although eight studies highlighted acceptance and validation by the participants’ intimate partners, spouses, siblings, and children (Baril & Silverman, 2024; Carvalho et al., 2024; Cashman, 2018; Cook-Daniels & Munson, 2010; de Vries et al., 2019; Fabbre & Gaveras, 2020; Gaveras et al., 2023; Willis et al., 2021), the participants in 11 studies shared experiences of estrangement from and rejection by family members and intimate partners (Baril & Silverman, 2024; Barsigian et al., 2020; Carvalho et al., 2024; Cook-Daniels & Munson, 2010; de Vries et al., 2019; Fabbre, 2017; Fabbre & Gaveras, 2020; Gallardo-Peralta et al., 2024; Knochel & Flunker, 2021; Lampe, 2024; Pang et al., 2019). For example, one participant in the United States recounted the pain of not being invited to a family member’s wedding (Fabbre & Gaveras, 2020), while a trans woman in Canada shared that her son had asked her to come as a man to attend his wedding (de Vries et al., 2019). Another trans woman in Canada shared that her daughter did not accept her identity and continued to call her “dad” (Baril & Silverman, 2024, p. 6). This rejection led the participants to fear that they would not have family members to care for them in their old age (Lampe, 2024; Pang et al., 2019). For example, a White, low-income, intersex, non-binary person shared the following: “I had my daughter […], but two years ago she became a born-again Christian and now hates [that] I’m non-binary and intersex […] It makes me uncertain about everything […] It makes me scared to have no one in my life who could take care of me during my final days” (Lampe, 2024, pp. 896–897).

The participants also reported experiences of being outed (Katz-Wise & Budge, 2015), pathologized, rejected, or discriminated against due to their gender identity within social groups, friendship circles, and religious communities (Bower et al., 2023; Fabbre & Gaveras, 2020; Siverskog, 2014). Some described feelings of social isolation after coming out (Siverskog, 2014) and frustration at having to constantly educate others about their identity (Löf & Olaison, 2020). Within the broader LGBTQ+ community, the participants noted that they found it challenging to integrate into the predominantly youth-oriented culture, which they felt erased aging and lacked awareness of transgender issues (de Vries et al., 2019). Many participants reported that they found it difficult to connect with younger people (Thelly et al., 2025) or that they had experienced exclusion within the transgender and gender-diverse community (Betts, 2021; Reygan et al., 2022; Toze et al., 2023). However, some shared their experiences of support and validation of their gender identity, as well as access to mental health and healthcare resources, through community interactions and rituals (Adan et al., 2021; Banerjee & Rao, 2020; Carvalho et al., 2024; Fabbre, 2014; Jesperson & Wesley, 2023; Jihanian, 2013; Lampe et al., 2025; Pang et al., 2019; Siverskog, 2014; Willis et al., 2021). For example, a two-spirit trans woman in her 40 s had participated in a renaming ceremony within her Indigenous community, where she was given a traditional female name that affirmed her gender identity (Jesperson & Wesley, 2023).

Five articles reported negative mental and cognitive health outcomes resulting from social stigmatization of gender identity and expression and gender-based discrimination. These outcomes included an increased risk of depressive symptoms (White Hughto & Reisner, 2018), suicidal intentions and behaviors (Fabbre & Gaveras, 2020; Putney et al., 2018), memory decline (Lambrou et al., 2022), reduced self-esteem, and the development of self-hatred (Banerjee & Rao, 2020).

#### Community building and advocacy

In eight studies, the participants reported that they were actively involved in community building and advocacy (Balcerek et al., 2021; Kia et al., 2025). In particular, those who had transitioned later in life felt a strong desire to help other transgender and gender-diverse people by sharing their transition-related experiences and knowledge (Katz-Wise & Budge, 2015). Some of the participants also described serving as role models within their communities and empowering their younger peers (Carvalho et al., 2024, 2025; Fabbre & Gaveras, 2020; Fabbre et al., 2023; Walker et al., 2016). Having had to overcome major challenges during their late transition, these participants shared their personal stories of coming out late in life to inspire their younger peers to come out earlier (Carvalho et al., 2025; Fabbre & Gaveras, 2020). These older adults leveraged their lived experiences to guide and support younger generations.

### Medical domain

Two subthemes were identified within the medical domain. The first subtheme, “Gender-affirming hormones and surgeries,” highlights the participants’ experiences in accessing these treatments, as well as the health outcomes. The second subtheme, “Gender-affirming and non-affirming health services,” explores the participants’ experiences with gender-affirming and non-affirming health services. Some of the participants raised concerns about the lack of trans-friendly and age-friendly services in long-term care facilities.

#### Gender-affirming hormones and surgeries

Twenty-one studies explored the participants’ desires and experiences regarding access to gender-affirming hormones and surgeries. Some explained that undergoing gender-affirming surgeries allowed them to present their identified gender in nursing homes and long-term care facilities (de Vries et al., 2019), while some wished to undergo bottom surgery before entering a long-term care facility (Knochel & Flunker, 2021). Two studies discussed the participants’ decision-making processes with respect to accessing gender-affirming hormones and surgeries (Jesperson & Wesley, 2023; Lampe, 2022). For instance, a White, lower-middle-class, bisexual trans woman living in the United States shared that the death of her spouse prompted her to re-evaluate her life and, ultimately, to decide to pursue medical affirmation (Lampe, 2022).

Although some individuals in the United Kingdom reported successfully navigating mental health gatekeepers and accessing gender identity clinics (Willis et al., 2020), many participants in Sweden, the United States, Canada, and the United Kingdom faced major barriers to accessing gender-affirming hormones and surgeries. These barriers included financial difficulties (Edwards et al., 2023), shortages of hormone replacement therapy (HRT), and the postponement of surgeries during the COVID-19 pandemic (Toze et al., 2023). Additional barriers included gatekeeping practices such as heteronormative restrictions (e.g., the requirement to divorce) (Hurd & Mahal, 2025) and agist attitudes among healthcare providers. Many participants were denied access to gender-affirming surgeries or hormones due to their age or health status (Baril & Silverman, 2024; Lampe, 2024; Siverskog, 2014, 2015). One trans woman in Sweden reported being questioned by a healthcare professional about whether gender-affirming surgery was “really necessary,” given that she had “so little time left to live” (Siverskog, 2014, p. 393). Other non-binary participants were denied access because they did not fit into binary categories (Siverskog, 2014; Toze & Westwood, 2025).

Although some of the participants raised health concerns about starting gender-affirming HRT at an advanced age (Willis et al., 2021) or worried about “losing time” on their journey (i.e., feeling they did not have “years and years to wait”; Willis et al., 2020, p. 1235), others challenged age-related biases of being “too old” for a medical transition, as highlighted by a British qualitative study in which 14 of the 22 transgender participants underwent gender-affirming hormone therapy and surgeries in their 40 s, 50 s, and 60 s (Fabbre, 2014, p.167; Willis et al., 2021). The participants who had access to medical affirmation reported that observing changes in their body brought them happiness (Carvalho et al., 2024).

Six articles reported health-related outcomes associated with the use of gender-affirming hormones. A quantitative study of 92 transgender and gender-diverse people aged 60 and older in the United States found that undergoing gender-affirming medical treatment was associated with higher quality-of-life scores (Cai et al., 2019). Another quantitative study of 71 transgender women aged 50 and older, referred to a national gender identity clinic service in the United Kingdom for assessment, found that transwomen who had used self-prescribed hormones prior to referral were significantly less anxious than their peers who had not used hormones (Bouman et al., 2016). One study noted a change in sex drive (Cook-Daniels & Munson, 2010), while another described the case of a transgender woman who developed an addiction to hormones in later life (Cashman, 2018). One trans woman in the United Kingdom reported menopausal symptoms following a reduction or cessation of HRT, which can cause hot flashes and night sweats, affect concentration, and is often associated with a lack of sleep (Toze & Westwood, 2025). Another study of transgender and gender-diverse people in Australia found that participants aged 45 and older were more likely than their younger counterparts to have been treated with transdermal estradiol and to have received lower doses of oral estradiol. However, the proportion of participants with a history of dyslipidemia, a cardiometabolic risk factor, was higher among those treated with transdermal estradiol than among those treated with oral estradiol (Balcerek et al., 2021). In summary, although the use of gender-affirming hormones is associated with positive psychological outcomes, such as enhanced quality of life and reduced anxiety, the literature also highlights potential health concerns in middle age and late life, including addiction, menopausal withdrawal symptoms, and a cardiometabolic risk factor.

#### Gender-affirming and non-affirming health services

Twenty-seven articles explored the participants’ experiences with gender-affirming and non-affirming health services. Seven of these articles highlighted positive experiences with non-judgmental and supportive professionals, including general practitioners and counselors who validated the participants’ gender identity and expression (Adan et al., 2021; Gaveras et al., 2023; Lampe, 2024; Lampe et al., 2025; Willis et al., 2021). Some articles also reported cases where professionals supported the participants in their medical gender affirmation journey later in life (Cook-Daniels & Munson, 2010; Fabbre & Gaveras, 2020; Lampe et al., 2025).

Other studies documented systemic gaps in the availability of gender-affirming and age-friendly services for transgender and gender-diverse middle-aged and older adults (Adan et al., 2021; Sharek et al., 2015). The key deficiencies included healthcare systems that prioritized physical health over gender-affirming needs (Baril & Silverman, 2024); providers who lacked competency in sexual health services and knowledge of medical gender affirmation (Lampe, 2024; Lampe & Pfeffer, 2024; Toze & Westwood, 2025; Williams et al., 2022); and compounding age- and health-related barriers, such as a lack of affordable mobility aids (e.g., motorized vehicles), which hinder older participants’ access to gender-affirming care (Guest et al., 2026). Many participants experienced gender-based discrimination, such as misgendering, deadnaming, mispronouncing (Baril & Silverman, 2024; de Vries et al., 2019; Faas et al., 2022; Guest et al., 2026; Hurd & Mahal, 2025; Knochel & Flunker, 2021), marginalization, and denial of care (Banerjee & Rao, 2020; Cook-Daniels & Munson, 2010; Fabbre et al., 2023; Kia et al., 2025; Pang et al., 2019). Some even suffered humiliating treatment, including not being allowed to shave (Faas et al., 2022) or being left “with breasts exposed on a gurney” (de Vries et al., 2019, p. 371).

Some of the participants expressed fears and concerns about the lack of trans-friendly services in long-term care facilities and nursing homes (Adan et al., 2021; Kortes-Miller et al., 2018; Lampe, 2024; Siverskog, 2014; Toze et al., 2023; Walker et al., 2023; Willis et al., 2021; Witten, 2015). Their concerns included rejection or discrimination by healthcare providers (Toze et al., 2023; Walker et al., 2023), being outed to staff and other residents in long-term care settings (Willis et al., 2021), and the risk of mistreatment as they aged (Adan et al., 2021; Witten, 2015). One participant worried about communicating their “non-transition” status to care workers, noting that these workers often seemed uncertain about how to categorize and respond to the needs of transgender and gender-diverse people who have not medically transitioned (Willis et al., 2021, p. 2806).

Several studies highlighted the participants’ concerns about age-specific vulnerability in healthcare settings (Adan et al., 2021; Knochel & Flunker, 2021; Pang et al., 2019; Putney et al., 2018; Walker et al., 2023; Witten, 2015). Some of the participants feared they would face gender-based discrimination if they found themselves in a state of “diminished capacity” and unable to advocate for themselves while receiving later- and end-of-life care (Pang et al., 2019, p. 49). Others feared losing their autonomy due to dementia and potentially seeing their gender identity erased (Adan et al., 2021; Witten, 2015). Some of the participants feared being involuntarily outed by care workers if they lost their autonomy as they aged (Walker et al., 2023). Finally, others expressed concerns about the ability of healthcare providers to respond with the necessary flexibility and competency should they develop cognitive impairments, particularly if they “regressed” to a time when they were not out of the closet or identified with their assigned gender at birth (Putney et al., 2018, p. 896).

### Legal domain

Two subthemes were identified within the legal domain. The first subtheme, “Navigating legal and institutional recognition of gender identity,” highlights the participants’ desire to obtain legal recognition of their gender and their experience of navigating institutional systems using their legal documents. The second subtheme, “Gender-affirming strategies in end-of-life documents,” encapsulates the participants’ efforts to legally protect their gender identity through end-of-life planning, as well as the systemic barriers that hinder these efforts.

#### Navigating legal and institutional recognition of gender identity

Six studies reported the participants’ attitudes toward or experiences of changing their legal names and/or gender markers, including their desire to obtain legal recognition of their gender, defined as laws and administrative procedures that enable them to update their legal identity documents to claim their identified gender (Arístegui et al., 2017; Barsigian et al., 2020; Carvalho et al., 2024; Hurd & Mahal, 2025; Lampe et al., 2025; Wolter et al., 2025). The participants who had gone through a long journey from identity suppression to gender affirmation, especially in middle age and late life, wished to live fully according to their desired gender identity, which included legal recognition of their gender (Carvalho et al., 2024). For example, the three transgender women in Carvalho et al.’s (2025) study shared the desire to be legally recognized as women, with possession of an official document helping to “prove what [she] already [is]” (p. 6). Following the implementation of Argentina’s Gender Identity Law in 2012, many Argentinean transgender women reported increased visibility and social acceptance (Arístegui et al., 2017). Some older transgender women, having lived their entire lives with their name and gender, feared that updating their legal documents would erase their past; for example, a 51-year-old participant explained: “We fear losing our identity, what will happen to our documents? What will happen with our things?” (Arístegui et al., 2017, p. 450).

One American study highlighted the positive experiences of the participants who received professional assistance in changing their legal name and gender marker at a time when this change was controversial (Lampe et al., 2025). Conversely, studies conducted in the United States, Canada, and Indonesia highlighted that the participants faced heteronormative barriers, restrictions, and identity erasure within institutional systems (Barsigian et al., 2020; Hurd & Mahal, 2025; Wolter et al., 2025). Even when the participants had updated their gender markers on their legal documents, some healthcare providers continued to deny their gender identity (Barsigian et al., 2020). Similarly, genderqueer participants described the challenges in navigating binary assumptions when filling out standard medical forms. For example, as a 40-year-old participant shared: “Because my driver’s license says M, my insurance says M, but really I was born female. These questions I have to answer about my prostate aren’t useful for us” (Barsigian et al., 2020). Beyond healthcare settings, institutional barriers were also evident in access to government services and social security programs. For example, transgender women in Indonesia faced discrimination, such as being called transphobic/homophobic terms, being asked to remove their makeup, or being prohibited from maintaining a gender-affirming appearance, when interacting with government services (Wolter et al., 2025). Furthermore, the challenges faced by these older participants were exacerbated by the Indonesian government’s social and economic support system, which only legally recognizes those who conform to traditional nuclear family structures and excludes older transgender women, who often live alone, are not married, and have no children. As a result, many older transgender women could not be included in state databases as welfare recipients (Wolter et al., 2025).

#### Gender-affirming strategies in end-of-life documents

Four studies highlighted the concerns, strategies, and barriers faced by the participants regarding end-of-life documents. A primary concern was the erasure of their gender identity in the absence of legal protections. As a 71-year-old trans man explained: “I worry that they will immediately after I am gone… start assuming that I am not who I was. They might decide that I am some girl” (Adan et al., 2021, p. 338). Consequently, some of the participants had resorted to gender-affirming strategies in legal documentation. One study reported that the participants had completed end-of-life documents to prevent the erasure of their identified gender (de Vries et al., 2019), while another noted that the participants had formalized advance care planning documents to ensure legal protection (Lampe, 2024). However, barriers to advance care planning, such as financial costs and lack of knowledge, were reported (Lampe, 2024). Another study conducted in several countries (e.g., the United States, Canada, Thailand) reported that the participants were not prepared for legal issues related to social and health services later in life (Witten, 2015).

## Discussion

This scoping review is one of the first to map the literature on the diverse experiences of gender affirmation among transgender and gender-diverse middle-aged and older adults, as well as cases where such affirmation is absent. Nearly half of the included studies were published after 2022, indicating a growing interest in this marginalized community. However, the literature largely focuses on the Global North and frequently relies on overlapping datasets and recurring research teams, with participants predominantly being transgender women and/or identifying as White. Consequently, the current literature lacks sufficient data to identify meaningful differences across varying geographical or sociocultural contexts. This skewed distribution has important implications for future research, calling for studies to explore gender-affirming experiences among individuals from low-income countries and ethnic minorities.

Although gender-affirming experiences are increasingly documented, the literature focuses primarily on how heteronormative and cisnormative structures create barriers for those seeking affirmation. Consequently, current research predominantly highlights non-affirming or invalidating experiences, resulting in a shortage of literature on positive gender-affirming experiences in the four domains. In the psychological domain, some transgender and gender-diverse people in middle age and late life continued to suppress their gender identity and/or internalize anti-transgender stigma. Research also highlights gender non-affirmation in the social domain, manifesting as rejection within interpersonal networks, forcing transgender and gender-diverse middle-aged and older individuals to manage and express their identity strategically. In the medical domain, research reveals a persistent denial of access to gender-affirming care and a lack of trans-inclusive and age-friendly services in long-term care facilities. In the legal domain, access to legal recognition of gender remains restricted and research on legal gender affirmation remains limited. This scarcity was also noted in the scoping review by King and Gamarel (2021) on transgender and gender-diverse populations, which included only four studies measuring legal gender affirmation. This lack of literature probably reflects the fact that only a handful of countries allow transgender and gender-diverse people to legally change their name and gender, and that many impose sterilization as a prerequisite. This gap also highlights the limited scholarly attention paid to transgender and gender-diverse older people in the legal domain; nevertheless, the lack of research does not diminish its importance for transgender and gender-diverse middle-aged and older adults. In fact, our review indicates that having their gender identity legally recognized and protected is important for their well-being and authenticity. This review provides compelling evidence regarding their desires, experiences, and barriers to accessing gender affirmation in four domains, encouraging practitioners and policymakers to better understand this field of research to develop policies and interventions.

The gender-affirming experiences of transgender and gender-diverse people in middle age and late life can be interpreted through the perspective of life course theory, a framework that considers the interplay of the timing of social roles and events, linked lives, the historical socio-cultural contexts in which these people live, and human agency (Elder, 1994; Fredriksen Goldsen et al., 2019; Witten, 2004). Our review reveals how *social timing*, which refers to the duration of roles and relevant expectations based on age, and *linked lives*, which refers to the interaction between an individual’s social worlds throughout their life (Elder, 1994), influence transgender and gender-diverse middle-aged and older adults’ gender affirmation. The participants’ social roles as breadwinner, parent, spouse, and employee hindered their decision to come out (Edwards et al., 2023; Fabbre, 2017). Coming out in middle age and late life also imposed unique challenges, such as shrinking social networks, loss of past relationships, or increased financial difficulties due to retirement or unemployment (Walker et al., 2016). The combination of isolation and reduced social or economic resources exacerbated the vulnerability of those who did not feel supported. Some expressed vulnerabilities and concerns, especially regarding old age, such as lack of social support, agist gatekeeping when seeking gender-affirming care, loss of autonomy, or exposure to transphobia and agism in healthcare settings, particularly in cases of cognitive decline (Adan et al., 2021).

The life course perspective highlights human agency, cultivated throughout their lives. Several participants demonstrated their agency and resilience in combating transphobia and agism to pursue gender affirmation. Middle age and late life offered them the opportunity to reimagine and affirm their identity. Some described how age-related bodily changes, such as menopause, affirmed their gender identity. Others experienced aging as a catalyst for gender exploration, self-acceptance, and self-actualization, ultimately achieving a sense of liberation. From this perspective, life events that can be considered traumatic and stressful, such as divorce (Willis et al., 2021), the death of parents (Fabbre, 2017) or spouse (Lampe, 2022), marital difficulties (Fabbre, 2017), and retirement (Siverskog, 2014), could simultaneously constitute turning points and opportunities for self-discovery. This perspective encourages the participants to reconstruct their identity through various pathways, whether it be coming out, pursuing medical affirmation, or embracing authenticity later in life. Such identity changes align with the life course principle of linked lives, as relational changes (e.g., divorce) often make room for personal growth and identity development, consistent with previous findings (Fredriksen Goldsen et al., 2019).

Although gender-affirming and non-affirming experiences are likely to be influenced by age groups and birth cohorts, a closer analysis of these differences was beyond the scope of this review. Many of the included studies covered broad age ranges without disaggregating the findings by age (e.g., de Vries et al., 2019). Nevertheless, we identified some age-specific nuances, although many of them spanned a wide range of ages. For instance, psychologically, the participants in their 50 s found that menopause facilitated their gender affirmation (Toze & Westwood, 2025; Xin & Lane, 2025). Socially, those in their 50 s to 70 s mentored their younger peers and encouraged them to come out earlier (Carvalho et al., 2024, 2025; Fabbre & Gaveras, 2020; Fabbre et al., 2023; Walker et al., 2016). Medically, the participants in their 60 s reported being denied access to gender-affirming surgeries or hormones due to their age or health status (Baril & Silverman, 2024; Lampe, 2024; Siverskog, 2014, 2015). Legally, although the participants in their 40 s noted that updating their legal documents could reduce police harassment, those in their 50 s and 60 s feared that changing these documents would erase their past (Arístegui et al., 2017). Future research is needed to examine these age-specific experiences in different age groups to develop tailored support for transgender and gender-diverse adults at all stages of middle age and late life.

The literature also suggests that the lived experiences of transgender and gender-diverse older adults are closely linked to their historical and social contexts (Fredriksen-Goldsen et al., 2014). Historical effects on the life course often manifest as cohort effects, where social changes at the macro level differentiate the life patterns of successive generations (Elder, 1994). For example, research conducted among American populations has divided transgender and gender-diverse older adults into the Invisible generation (born before 1934), the Silenced generation (born between 1935 and 1949), and the Pride generation (born between 1950 and 1964), thus reflecting cohort differences in community engagement and experiences of discrimination (Fredriksen-Goldsen, 2016; Fredriksen-Goldsen et al., 2024). While we recognize that middle-aged and older adults represent distinct birth cohorts, the data extracted from our review were insufficient to analyze gender-affirming experiences across cohorts. Because the studies reviewed largely aggregated these age groups, we could only observe shared experiences among people born in earlier decades, who had to navigate historical periods devoid of affirming language, social resources, and institutional recognition. This context, compounded by anti-LGBTQ+ social stigma, often prolonged their journey toward self-acceptance, coming out, and an authentic life (Barsigian et al., 2020; Edwards et al., 2023). Furthermore, the historical inaccessibility of gender-affirming services, the absence of gender recognition laws, and restrictive medical prerequisites (e.g., the requirement of medical diagnoses or sterilization) hindered their access to medical and legal affirmation (Fabbre & Gaveras, 2020). Given these cohort differences, future research should consider transgender and gender-diverse middle-aged and older adults a heterogeneous group, investigating the role played by specific historical events in their gender affirmation journeys. Furthermore, although our study has important implications for the participants involved in the studies reviewed, we must be cautious and not overgeneralize these findings to younger cohorts who will eventually age. Because the experience of aging is shaped by historical context, future research and interventions should contextualize the unique cohort effects of subsequent generations.

Our review indicates that not all transgender and gender-diverse people have equitable access to their desired gender affirmation (Lacombe-Duncan et al., 2025), as this access is often contingent on their social position and the availability of financial, social, medical, and legal resources. Although the included studies primarily focused on the intersection of age and transgender or gender-diverse identity, the participants’ experiences are intricately shaped by other social identities. Characteristics such as religious affiliation (Bower et al., 2023; Fabbre et al., 2023; Reygan et al., 2022), intersex variations (Lampe, 2024), race and ethnicity (Cashman, 2018), sexual minority status (Witten, 2015), and socioeconomic or geographical situation (Baril & Silverman, 2024; Wolter et al., 2025) intersect to create vulnerabilities or viabilities in the process of gender affirmation. For instance, among lesbian participants, the intersection of multiple marginalized social identities (e.g., advanced age, transgender female identity, and sexual minority status) exposed them to compounding systems of oppression—agism, transphobia, and heterosexism—which exacerbated systemic barriers to accessing gender-affirming care (Witten, 2015). Conversely, White people with college degrees often benefit from privileges that facilitate their access to medical, social, and legal recognition and resources compared with less educated people of color (Bower et al., 2023; Guest et al., 2026; Scheim et al., 2020; Williams et al., 2022). Because these factors are rarely examined systematically in the literature, we recommend intersectionality as a critical lens for future research. This intersectional lens, which examines how social categories (e.g., gender identity, sexual orientation, and race) interact with social systems of privilege and disadvantage to situate and influence individuals and communities (Collins et al., 2019; Crenshaw, 1991), offers a valuable framework for interpreting participants’ gender-affirming experiences. Future research should adopt an intersectional perspective to examine how these social identities interact and influence gender-affirming experiences.

## Recommendations for future practice

Based on our findings, we make the following recommendations for developing age-friendly and trans-affirming programs. We suggest setting up peer support groups that are adapted and rooted in the community. As the included studies highlight, these community networks not only provide essential psychological validation but also offer practical assistance to help older adults navigate the multifaceted barriers to accessing gender-affirming care (Lampe et al., 2025). Furthermore, our review indicates that many older participants actively seek to mentor, advocate for, and share their gender affirmation narratives with younger individuals. Therefore, developing intergenerational programs involving young, middle-aged, and older adults can foster community building (Bietsch, 2026).

Gender affirmation in the medical domain involves the provision of gender-affirming care and the presence of competent healthcare providers. In healthcare settings, it is recommended to implement comprehensive and trans-inclusive training, with a specific focus on gender clinics and long-term and end-of-life care facilities. Ideally, such training should be developed in collaboration with the transgender and gender-diverse community and monitored over time. Research shows that trans-inclusive training prepares healthcare professionals to provide competent and sensitive care, which can enhance the quality of care experienced by transgender and gender-diverse individuals (Jecke & Zepf, 2024). Research suggests partnering with non-governmental organizations to organize trans-inclusive care training for healthcare professionals (Blus-Kadosh & Hartal, 2025). Additionally, developing culturally sensitive guidelines can promote best practices and ensure that geriatric care professionals are prepared to meet the needs of transgender and gender-diverse middle-aged and older adults (Bass & Nagy, 2026), thereby preventing misgendering, involuntary outing, and non-affirmation.

At the legal level, lawmakers must recognize that legal recognition of gender (including recognition of non-binary people) is a key condition for access to basic rights and a protective factor for transgender health (Winter et al., 2016). To mitigate administrative barriers, targeted training on transgender issues should be provided to public servants and legal professionals (Arístegui et al., 2017). We also recommend offering age-friendly administrative assistance to help transgender and gender-diverse older adults complete their advance care directives and end-of-life documents, while ensuring the protection of their gender identity.

## Limitations and implications for future research

This study has several limitations. First, regarding our search strategy, as we focused on peer-reviewed empirical research, we did not include gray literature, such as reports from community organizations or policy documents. Additionally, we did not involve a research librarian to ensure comprehensive coverage of the relevant literature. Future reviews should include gray literature and involve specialized research librarians. Second, this review was limited to articles published in English due to funding and time constraints, as well as a lack of expertise in languages other than English. As a result, it is subject to language and cultural biases and may have overlooked the views of transgender and gender-diverse individuals in non-English-speaking contexts. Third, the included studies had varying sample sizes and levels of representation of trans women, trans men, as well as non-binary and gender-diverse individuals; collapsing these distinct identities and experiences under the umbrella term “transgender and gender-diverse” in our synthesis risks leading to oversimplification. Future research should disaggregate participants and data to examine the unique trajectories of gender affirmation within different subgroups. In addition, trans women were predominant among the participants, highlighting the need for future research to prioritize non-binary, genderqueer, and other gender-diverse individuals.

## Conclusion

This review contributes to the growing body of literature aimed at providing an overview of gender-affirming (and non-affirming) experiences among transgender and gender-diverse individuals in middle age and late life. The needs and desires of these individuals regarding gender affirmation are crucial and should not be overlooked. Our findings reveal a significant gap in research, particularly regarding the legal dimension of gender affirmation and gender-affirming experiences (as opposed to non-affirming experiences) in the four key domains. This review provides practitioners and policymakers with evidence to promote age-friendly and trans-inclusive programs, as well as to strengthen legal protections.

## Supplementary Material

gnag194_Supplementary_Data

## Data Availability

This study does not contain primary datasets. The literature reviewed has been listed in the Supplementary Material.

## Preregistration

The reviews have been registered on https://osf.io/q8g3r.
