# Supplementary material for: Gender affirmation among transgender and gender-diverse people in middle age and late life: a scoping review
Source: Gerontologist. 2026 Aug 12;66(9):gnag194. doi: 10.1093/geront/gnag194 (PMC13541382; doi:10.1093/geront/gnag194)
Supplement: Supplementary file 1 [file gnag194_supplementary_data.docx]

# **Gender affirmation among transgender and gender-diverse people in middle age and late life: a scoping review**

# Appendix 1

Journal articles were searched in databases using the following keywords and search strings:

| **Database** | **Limitations** | **Concept 1: Transgender** |  | **Concept 2: Gender affirmation** |  | **Concept 3: Middle to Old adult** | **Search Findings (Date April 7)** |
| --- | --- | --- | --- | --- | --- | --- | --- |
| APA PsycINFO, ASSIA, and Sociological Abstracts from ProQuest | English, article | Transgender OR “trans men” OR “trans women” OR “gender non-conforming” OR “gender nonconforming” OR “gender minorities” OR genderqueer OR “gender queer” OR “non-binary” OR nonbinary | AND | “gender affirm*” OR “gender-affirm*” OR “gender congruence” OR “gender incongruence” OR self-affirmation OR “gender identity” OR transition OR “gender recognition” OR “legal name” OR “name change” OR “gender marker” OR “status change” OR documentation OR “hormon* therapy” OR HRT OR  “gender reassignment” OR “sex reassignment” OR “breast surgery” OR “chest surgery” OR vaginoplasty OR phalloplasty OR silicone OR “top surgery” OR “bottom surgery” OR  “voice therapy” OR “hair removal” OR muscle OR  “coming out” OR disclosure OR “social support” OR “family support” OR “peer support” OR “outness” OR “role casting” OR validat* OR invalidat* OR pronoun* OR pass* OR “physical activity” OR exercise* OR “gender expression” OR dress* OR “gender-neutral” OR infrastructure* OR facilities OR policy OR policies OR marriage OR “gender euphoria” OR “gender dysphoria” OR “body image*”OR diagnosis OR diagnostic OR assessment OR eating OR diet OR weight OR  “end-of-life” OR “end of life” OR “advance care planning” OR  “long-term care” OR healthcare OR “health care” OR “social care” OR “social service*” OR “social program*” OR “human service*” | AND | “aging” OR “older adult” OR “elderly” OR “elder” OR “late* life” OR  middle OR mid-life OR midlife OR “Mid-to-late life” OR “mid- to late-life” | 447 |
| MEDLINE, Social Work Abstracts, and CINAHL  PLUS from EBSCOhost | English, Peer-reviewed |  |  |  |  |  | 387 |
| Scopus | English, article |  |  |  |  |  | 495 |

# Appendix 2

Descriptive details of included papers.

| **Study no.** | **Author(s) and year** | **Country/ location** | **Aims of study** | **Sample size/characteristics** | **Gender-affirming experiences domains** | | | | **Study Design/Methods** | | | **Funding** |
| --- | --- | --- | --- | --- | --- | --- | --- | --- | --- | --- | --- | --- |
|  |  |  |  |  | **Psychological** | **Interpersonal/Social** | **Medical/ Healthcare setting (Professional)** | **Legal** | **Quantitative Method** | **Mixed Methods** | **Qualitative Method** |  |
| 1 | Adan et al., 2021 | USA | To identify the unique needs of the transgender elder community. | N = 19 Transgender  (Including N=10 transgender women, N=9 transgender men)  64-84 years  All white | X | X | X | X |  |  | X | No funding. |
| 2* | Arístegui et al., 2017 | Argentina | To explore the impact of the implementation of the Argentinean Gender Identity Law on the living conditions, plus it explored stigma and discrimination experiences of transgender women. | N=20 Transgender women  (Including N=11 aged 40+) N=4 Complete high school and above  N=7 Incomplete high school and below Age of transition: 11-20 |  |  | X | X |  |  | X | Open Society Foundations. |
| 3 | Balcerek et al., 2021 | Australia | To evaluate prescribing patterns of feminizing hormone in older (age ≥45) versus younger (age <45) trans individuals, in the context of cardiovascular risk factors that emerge with aging. | N=55 Transgender  45+ years |  |  | X |  | X |  |  | National Health and Medical Research Council Postgraduate Scholarship; Australian Government National Health and Medical Research Council Early Career Fellowship; Viertel Charitable Foundation Clinical Investigator Award, Endocrine Society of Australia, Austin Medical Research Foundation, and the Royal Australasian College of Physicians Foundation. |
| 4 | Banerjee & Rao, 2021 | India | To explore the lived experiences and psychosocial challenges of older transgender adults during the COVID-19 pandemic in India. | N=10 Transgender  60-75 years  (Including N=4 identified as "third gender", N=2 Male, N=1 Female, N=1 Prefer not disclose; N=6  Age at transition 18-30) | X | X | X |  |  |  | X | NA |
| 5 | Baril & Silverman, 2024 | Canada | To examine the unique aspects of living with dementia as a trans or non-binary person. | N=6 Total (Including N=2 trans people living with dementia, White, Urban, 75 years) | X | X | X |  |  |  | X | Social Sciences and Humanities Research Council of Canada. |
| 6 | Barsigian et al., 2020 | USA | To examine the generational differences in the lived experience of genderqueer people. | N=30 genderqueer sexual minorities (Including N=13 in mid- and late-life) | X | X |  | X |  |  | X | Eunice Kennedy Shriver National Institute of Child Health and Human Development. |
| 7 | Betts, 2021 | New Zealand | To report how access to social spaces is filtered through perceptions of who is wel- come in lesbian, gay, bisexual, transgender, and queer spaces. | N=31 LGBTQ+  60-80 years  (extracted data from N=2 Transgender, 60-68 years) |  | X |  |  |  |  | X | NA |
| 8* | Bouman et al., 2016 | UK | To 1) describe the sociodemographic and clinical features of trans people over the age of 50 years referred to a national gender identity clinic service during a 30-month period. 2) collect and analyze the use and the source of CHT prior to referral to a gender identity clinic service, and 3) to investigate the influence of CHT on psychopathology | N=71 Transfemale  50+ years |  | X | X |  | X |  |  | No funding. |
| 9 | Bower et al., 2023 | USA | To explore the processes of spiritual reconciliation from the perspective of LGBTQ older adults. | N=13 LGBTQ  46-76 years  (Including N=1 57-year-old Transgender man; N=1 63-year-old Transgender woman) All White | X | X |  |  |  |  | X | No funding. |
| 10* | Cai et al., 2019 | USA | To examine whether older age moderates the association between gender-affirming medical treatment and quality of life (QOL) among transgender individuals. | N=2420 Transgender  18+ years  (Including N=92 60+ years) Predominantly White |  |  | X |  | X |  |  | National Institute on Minority Health and Health Disparities grant; National Institute on Aging grant. |
| 11 | Carvalho, Casado, et al., 2024 | Portugal and Spain | To identify and characterize the intended legacies of transgender women from Spain and Portugal who embarked on their gender transition at 40+ years. | N=7 Transgender women (Including N=3 Portuguese and N=4 Spanish) 52-66 years  Age of transition: 40s-64 | X | X |  |  |  |  | X | National funds through Fundação para a Ciência e a Tecnologia (FCT). |
| 12 | Carvalho, Tavares, et al., 2024 | Portugal | To explore the life trajectories of three Portuguese transgender women who transitioned later in life (50+ years old) by identifying key chapters in their life courses. | N=3 Portuguese transgender women 56-65 years | X | X | X | X |  |  | X | National funds through Fundação para a Ciência e a Tecnologia (FCT). |
| 13 | Cashman, 2018 | USA | To examine the interconnectedness of coming out, migration, physical transformation (hormones, gender confirmation surgery), and ageing, with a particular focus on the indexing of time, space and transition. | N=1 Transwoman  50 years |  | X | X |  |  |  | X | NA |
| 14 | Cook-Daniels & Munson, 2010 | USA | To report findings about sexual violence, elder abuse, and sexuality on transgender adults age 50 years and older. | FORGE Survey sample  N=302 transgender people and Significant Others (Including N=53 Transgender people  Aged: 50+)   FORGE’s Transgender Aging Network N=56 people   FORGE’s Survey on Sexuality of Transgender Elders N=829 Transgender people and/or their partners  (including N=272 Transgender people Aged 50+) | X | X | X |  |  | X |  | Wisconsin AIDS Fund; Johnson and Pabst LGBT Humanity Fund. |
| 15 | de Vries et al., 2019 | Canada | To explore end-of-life concerns among LGBT older adults | N=93 LGBT  55-89 years  (Including N=24 Transgender) |  | X | X | X |  |  | X | Canadian Frailty Network. |
| 16 | Edwards et al., 2023 | USA | To employ an intersectionally-informed strengths-based, con- structivist grounded theory approach to explore joy and positive SGM identity, both conceptu- alized as major contributors to coping and resilience among SGM people. | N=315 Sexual and gender minority (Including N=77 Gender-expansive, N=39 Transfeminine, N=36 Transmasculine) 18+ years  Predominantly White (Data extracted from N=3 Transgender, aged 50-65) | X |  | X |  |  |  | X | PatientCentered Outcomes Research Institute. |
| 17 | Faas et al., 2022 | USA | To explore the experiences and issues of older LGBTQ+ adults in the US, in the context of the COVID-19 pandemic. | LGBTQ+ Sample size and age unspecified  (Data extracted from N=1 older White transwoman) |  |  | X |  |  |  | X | NA |
| 18 | Fabbre, 2014 | USA | To interpret narratives about gender transitions in later life by integrating these two perspectives and generating implications for gerontological social work. | N=22 Male-to-female  50-82 years | X | X | X |  |  |  | X | Center on Aging at the University of Chicago through a National Institute on Aging Training Grant |
| 19 | Fabbre, 2015 | USA | To explore the experiences of transgender persons who contemplate or pursue a gender transition in later life in order to develop culturally diverse conceptualisations of health and wellness in older age. | N=22 Male-to-female  50-82 years | X | X |  |  |  |  | X | Center on Aging at the University of Chicago through a National Institute on Aging Training Grant |
| 20 | Fabbre, 2017 | USA | To explore the conditions under which people contemplate or pursue a gender transition in later life. | N=22 Male-to-female 50-82 years | X | X |  |  |  |  | X | National Institute on Aging at the National Institutes of Health;  Center for Health Administration Studies at the University of Chicago |
| 21 | Fabbre & Gaveras, 2020 | USA | To explore how multilevel stigma is experienced by transgender and gender nonconforming older adults, who navigate stigmatizing environments over a lifetime and who may be especially vulnerable to its cumulative effects. | N=88 Transgender and gender nonconforming people 50-90 years | X | X | X |  |  |  | X | Brown School at Washington University in St. Louis; National Institute of Mental Health. |
| 22 | Fabbre et al., 2023 | USA | To explore the meaning of spirituality in the lives of trans older adults. | N=88 transgender and/or gender nonconforming people  50-90 years | X | X | X |  |  |  | X | National Institute of Mental Health. |
| 23 | Gallardo-Peralta et al., 2024 | Chile | To understand how different life courses have influenced the structuring of social networks in old age among gay men and trans women in Chile. | N=13 LGBT (Including N=3 trans women, 53-70 years) |  | X |  |  |  |  | X | Agencia Nacional de Investigacion y Desarrollo (ANID, Chile) FONDECYT Regular. |
| 24 | Gaveras et al., 2023 | USA | To illuminate the lived experiences of suicidal ideation and behavior in older transgender adults. | N=14 Transgender 50-70 years | X | X | X |  |  |  | X | National Institute of Mental Health. |
| 25 | Guest et al., 2025 | USA | To explore the barriers and needs of LGBTQ older adults. | N=672 LGBTQ  50+ years  (Majority identified as White gay men and lived in urban environment)  extracted data from N=2 transgender, N=1 nonbinary transgender |  |  | X |  |  |  | X | JustFundKY, The University of Kentucky Donovan Scholarship in Gerontology, the University of Kentucky James S. Brown Award, and the University of Kentucky Office of LGBTQ* Resources; NIH National Center for Advancing Translational Sciences. |
| 26 | Hurd & Mahal, 2025 | Canada | To explore older LGBTQ+ Canadians' experiences of prejudice and discrimination. | N=30 LGBTQ+  65-83 years  (Including N=5 Transgender Women N=1 Transgender Man) |  | X | X | X |  |  | X | Social Sciences and Humanities Research Council of Canada Insight Grant. |
| 27 | Jesperson & Wesley, 2023 | Canada | To explore a life story of a two-spirit elder woman. | N=1 two-spirit transwoman | X | X | X |  |  |  | X | NA |
| 28 | Jihanian, 2013 | USA | To address the question “What does it mean for long-term care providers to be responsive to lesbian, gay, bisexual, and transgender (LGBT) older adults?”. | N=3 LGBT (Interview)  Including N=1 transgender woman  64-74 years   N=4 Gay men (Focus group) 61-79 years   All White. |  | X |  |  |  |  | X | NA |
| 29 | Katz-Wise & Budge, 2015 | USA | To investigate transgender women's emotional experiences and cognitive and interpersonal identity processes related to gender transitioning in mid-life | N=13 Transgender women 44-67 years   (Including N=11 White  N=2 White/Native American) | X | X |  |  |  |  | X | NA |
| 30 | Kia et al., 2024 | Canada | To examine the perspectives of different participant groups to develop insight into TGD older adults’ issues and priorities in the context of their engagements with systems of care. | N=21 Transgender and gender-diverse adults and service providers  (Including N=10 Transgender and gender-diverse adults, 51-73 years)  Predominantly woman,White |  | X | X |  |  |  | X | Social Sciences and Humanities Research Council. |
| 31 | Knochel & Flunker, 2021 | USA | To explore how gender identity affects transgender and nonbinary older adults’ fears, hopes, and plans for use of institutional long-term care. | N=24 Transgender and nonbinary  56-73 years, average 63  81.82% identified as MTF, transwoman, female, or MTF transwoman, while 18.18% identified as a transguy, transgender man, or FTM.  Predominantly White. |  | X | X |  |  |  | X | University of Minnesota Clinical and Translational Science Institute;  National Center for Advancing Translational Sciences of the National Institutes of Health. |
| 32 | Kortes-Miller et al., 2018 | Canada | To examine hopes and fears of older LGBTQ+ adults considering long-term care as they face end of life. | N=23 LGBT 57-78 years  (Sample size of transgender unspecified, data extracted from N=1 Transgender) |  |  | X |  |  |  | X | NA |
| 33 | Lambrou et al., 2022 | USA | To explore the psychosocial factors associated with subjective cognitive decline in a sample of TNB older adults. | N=115 Transgender or non-binary  50-76 years  (Including N=52 Transgender women  N=23 Transgender men N=22 Men  N=40 Genderqueer/ Nonbinary  N=14 Another gender identity) Mostly White.   N=592 Cisgender LGBQ+  50-82 years |  | X |  |  | X |  |  | National Institutes of Health, National Institute on Aging. |
| 34 | Lampe, 2022 | USA | To explore how transgender and non-binary older Americans manage their health during the COVID-19 pandemic. | N=47 Transgender and non-binary older people  65+ years (N=27 Transgender women  N=1 Transsexual woman  N=10 Transgender men  N=1 Transgender  N=3 Non-binary N=1 Blended- gender  N=1 Pangenderfluid N=2 Two-spirit  N=1 Questioning)  Mostly White, middle classs |  |  | X |  |  |  | X | The University of South Carolina; National Institute on Aging of the National Institutes of Health. |
| 35 | Lampe, 2024 | USA | To examine how transgender older adults perceive and plan for ageing and end-of-life experiences through advance care planning. | N=47 Transgender and non-binary older people  65+ years (N=27 Transgender women  N=1 Transsexual woman  N=10 Transgender men  N=1 Transgender  N=3 Non-binary N=1 Blended- gender  N=1 Pangenderfluid N=2 Two-spirit  N=1 Questioning)  Mostly White, Middle classs |  | X | X | X |  |  | X | The University of South Carolina; National Institute on Aging; Alzheimer’s Association. |
| 36 | Lampe & Pfeffer, 2024 | USA | To examine how Transgender, non-binary, and intersex people older adults experience and mitigate inequity in sexual and reproductive healthcare. | N=50 Transgender, non-binary, and intersex people (N=27 Transgender women  N=1 Transsexual woman  N=10 Transgender men  N=1 Transgender  N=3 Non-binary N=1 Blended- gender  N=1 Pangenderfluid N=2 Two-spirit  N=1 Questioning) 65+ years |  |  | X |  |  |  | X | The University of South Carolina; National Institute on Aging; Alzheimer’s Association. |
| 37 | Lampe et al., 2025 | USA | To investigate facilitators contributing to positive or satisfactory behavioral healthcare experiences among a sample of transgender and gender nonconforming older adults. | N=47 Transgender and gender nonconforming people (N=27 Transgender women  N=1 Transsexual woman  N=10 Transgender men  N=1 Transgender  N=3 Non-binary N=1 Blended- gender  N=1 Pangenderfluid N=2 Two-spirit  N=1 Questioning) 65-81 years |  | X | X | X |  |  | X | The University of South Carolina; National Institute on Aging; Alzheimer’s Association. |
| 38 | Löf & Olaison, 2020 | Sweden | To explore how older Swedish LGBTQ adults reason about openness in an elder care context concerning their future needs for services and adopts Nancy Fraser’s theoretical framework of recognition. | N= 15 LGBTQ  65+ years  (Including N=5 transgender) |  | X |  |  |  |  | X | NA |
| 39 | Miedema et al., 2022 | Thailand | To evaluate whether and how gender identity, independent of and together with same-sex sexuality, influences experiences of stigma among gender non-conforming sexual minority women. | N=21 Toms 20-43 years  Mostly identified neither men nor women)  (Data extracted from N=1 43-year-old participant) |  | X |  |  |  |  | X | Mount Holyoke Alumnae Research Grant; Emory University. |
| 40 | Pang et al., 2019 | Canada | To explore planning for later and end-of-life care among transgender older adults. | N=24 Transgender  Mean: 70 years |  | X | X |  |  |  | X | Canadian Frailty Network, formerly Technology Evaluation in the Elderly. |
| 41 | Putney et al., 2018 | USA | To explore the anticipated needs of LGBT older adults in residential care, particularly those in suburban and rural areas | N=50 LGBT 55-87 years  (Including N=3 transgender) |  | X | X |  |  |  | X | Simmons College President’s Fund for Faculty Excellence. |
| 42 | Reygan & Henderson, 2019 | South Africa | To explore the lived experiences of LGBT people over 50 years of age in the Western Cape and Gauteng provinces of South Africa. | N= 22 LGBT (Including N=3 transgender, people of color) 50-72 years | X |  |  |  |  |  | X | National Research Foundation of South Africa. |
| 43 | Reygan et al., 2022 | South Africa | To explore the experiences of ageing and care among LGBT elders in South Africa. | N= 22 LGBT (Including N=3 transgender, people of color) 50-72 years |  | X |  |  |  |  | X | National Research Foundation of South Africa. |
| 44 | Sharek et al., 2015 | Ireland | To detail older LGBT persons’ usage, experiences and concerns with accessing healthcare services, disclosing their LGBT identity to professionals, preferences for care and their suggestions for improvement in services, including nursing services. | N=144 LGBT (Survey sample)  N=36 (Interview sample) 55+ years   (Including N=12 Transgender) |  |  | X |  |  | X |  | The Gay and Lesbian Equality Network (GLEN), Age & Opportunity and the Health Service Executive (HSE). |
| 45 | Siverskog, 2014 | Sweden | To utilize a biographical approach to explore how earlier life experiences matter in later life, and how age and (nonconforming) gender identities can be understood in relation to one another. | N=6 Transgender  62-78 years  (Including N=3 transsexual, N=1 man, N=2 woman, N=1 Genderqueer) All white | X | X | X |  |  |  | X | Swedish Research Council for Health, Working Life and Welfare; the Solstickan Foundation. |
| 46 | Siverskog, 2015 | Sweden | To explore intersections between (old) age, gender, and embodiment. | N=6 Transgender  62-78 years  (Including N=3 transsexual, N=1 man, N=2 woman, N=1 Genderqueer) All white | X | X | X |  |  |  | X | Swedish Research Council for Health, Working Life and Welfare; the Solstickan Foundation. |
| 47 | Thelly et al., 2025 | India | To explore lived experiences of elderly transgender individuals residing in trans communities in Pondicherry, with a focus on their challenges in accessing end-of-life care and the broader societal, economic, and intergenerational issues affecting their well-being. | N=6 Transgender  50+ years |  | X |  |  |  |  | X | NA |
| 48 | Toze et al., 2023 | UK | To explore older trans and gender non-conforming people’s social support networks, key concerns and unmet needs during the COVID-19 ‘lockdown’. | N=38 Trans and gender non-conforming people 60+ years  Including N=23 women |  | X | X |  |  | X |  | NA |
| 49* | Toze & Westwood, 2025 | UK | To explore experiences of menopause and accessing services and support within the trans, gender non-conforming and non-binary population. | N=15 Trans, gender non-conforming and non-binary (Including N=12 non-binary  N=1gender non-conforming  N=1 man  N=1 woman) 40-49 years  *All White. | X |  | X |  |  |  | X | No funding. |
| 50* | Walker et al., 2016 | USA | To explore one older transgender woman’s quest for identity and some of the challenges inherent in the phenomenon of transgender transitioning in later life. | N=1  Transgender female  70 years | X | X |  |  |  |  | X | The Beta Alpha Chapter of Sigma Theta Tau International and the Hogstel Gerontological Research Award. |
| 51 | Walker et al., 2023 | 24 countries (USA, Canada, Australia, Sweden. United Kingdom) | To understand concerns about receiving care in a sample of transgender, gender non- binary, and gender diverse adults across the lifespan. | N=829 Transgender and gender-diverse people 18-70 years  (Including N=572 in middle and older age, mostly White) |  |  | X |  |  | X |  | No funding. |
| 52 | White Hughto & Reisner, 2018 | USA | To investigate the relationship between discrimination and mental health in aging transgender adults | N=61 Transgender  50-75 years  (Including N=47 MTF  N=14 FTM) |  | X |  |  | X |  |  | The Miller Foundation; the National Institute of Mental Health; National Institute on Minority Health Disparities. |
| 53 | Williams et al., 2022 | USA | To explore how the how the rural south central Appalachian context intersects with the identities of LGBT+ older adults with regard to accessing formal services. | N=11 LGBT+  60-88 years  (Including N=1 Transgender Caucasian) |  |  | X |  |  |  | X | NA |
| 54* | Willis et al., 2020 | UK | To (1) examine supportive and obstructive points of interaction with health-care professionals, and (2) identify key learning messages for improving trans-related health care from the perspectives of trans-identifying adults in later life. | N=19 Transgender  50-74 years  (Including  N=15 Trans women  N=4 Trans men) All White | X |  | X |  |  |  | X | The Dunhill Medical Trust. |
| 55 | Willis et al., 2021 | UK | To explore older trans and gender non-conforming people's concerns for and expectations of later life. | N=22 Trans and gender non-conforming people  (Including N=10 Aged 50-59 N=12 Aged 60+  N=15 Trans women  N=4 Trans men N=2 Cross-dressers  N=1 Gender fluid)  All white | X | X | X |  |  |  | X | The Dunhill Medical Trust. |
| 56 | Witten, 2015 | 24 countries (USA, Canada, Australia, Sweden. United Kingdom) | To examine the experiences and needs of an international sample of current, English-speaking, lesbian, transgender-identified (trans-lesbian) adults around a number of later life and end-of-life perceptions, preparations, and concerns. | N=1,963 Transgender (Including N=276 Transgender Lesbian) 61.1% Aged 51+ years |  |  | X | X |  | X |  | NA |
| 57 | Wolter et al., 2025 | Indonesia | This article discusses older transgender women’s barriers to accessing government health and social welfare programs in Indonesia, and how intersecting factors like gender, age, and poverty contribute to poor health outcomes. | N=48 Transgender women (Survey sample)  (N=31 Aged 40-59  N= 11 Aged 60- 69 N=6 Aged 70+)  N=15 transgender women  N=5 government officials (Interview sample) |  |  |  | X |  |  | X | Indonesia AIDS Coalition. |
| 58 | Xin & Lane, 2025 | Australia | To explore the meanings and experiences of menopause for trans and gender diverse (TGD) people and how menopause affects clinical practice. | N=3 TGD community leaders  (Including N=1 55-year-old Transman  N=1 70-year-old Transwoman)  N=3 medical practitioners | X |  |  |  |  |  | X | No funding. |

Note: Studies marked with an asterisk were identified through other sources (Google Scholar or citation searching) rather than database/register searches.

**Preferred Reporting Items for Systematic reviews and Meta-Analyses extension for Scoping Reviews (PRISMA-ScR) Checklist**

| **SECTION** | **ITEM** | **PRISMA-ScR CHECKLIST ITEM** | **REPORTED ON PAGE #** |
| --- | --- | --- | --- |
| **TITLE** | | | |
| Title | 1 | Identify the report as a scoping review. | Yes; Title page |
| **ABSTRACT** | | | |
| Structured summary | 2 | Provide a structured summary that includes (as applicable): background, objectives, eligibility criteria, sources of evidence, charting methods, results, and conclusions that relate to the review questions and objectives. | Yes; Page 2 |
| **INTRODUCTION** | | | |
| Rationale | 3 | Describe the rationale for the review in the context of what is already known. Explain why the review questions/objectives lend themselves to a scoping review approach. | Yes; Page 3-6 |
| Objectives | 4 | Provide an explicit statement of the questions and objectives being addressed with reference to their key elements (e.g., population or participants, concepts, and context) or other relevant key elements used to conceptualize the review questions and/or objectives. | Yes; Page 5-6 |
| **METHODS** | | | |
| Protocol and registration | 5 | Indicate whether a review protocol exists; state if and where it can be accessed (e.g., a Web address); and if available, provide registration information, including the registration number. | No; PROSPERO website doesn’t allow registration for scoping review. We registered our study on OSF (see Page 7). |
| Eligibility criteria | 6 | Specify characteristics of the sources of evidence used as eligibility criteria (e.g., years considered, language, and publication status), and provide a rationale. | Yes; Page 7 |
| Information sources* | 7 | Describe all information sources in the search (e.g., databases with dates of coverage and contact with authors to identify additional sources), as well as the date the most recent search was executed. | Yes; Page 7 |
| Search | 8 | Present the full electronic search strategy for at least 1 database, including any limits used, such that it could be repeated. | Yes; Page 7, Appendix 1 |
| Selection of sources of evidence† | 9 | State the process for selecting sources of evidence (i.e., screening and eligibility) included in the scoping review. | Yes; Page 9 |
| Data charting process‡ | 10 | Describe the methods of charting data from the included sources of evidence (e.g., calibrated forms or forms that have been tested by the team before their use, and whether data charting was done independently or in duplicate) and any processes for obtaining and confirming data from investigators. | Yes; Page 8-10 |
| Data items | 11 | List and define all variables for which data were sought and any assumptions and simplifications made. | Yes; Page 9 |
| Critical appraisal of individual sources of evidence§ | 12 | If done, provide a rationale for conducting a critical appraisal of included sources of evidence; describe the methods used and how this information was used in any data synthesis (if appropriate). | NA |
| Synthesis of results | 13 | Describe the methods of handling and summarizing the data that were charted. | Yes; Page 9-10 |
| **RESULTS** | | | |
| Selection of sources of evidence | 14 | Give numbers of sources of evidence screened, assessed for eligibility, and included in the review, with reasons for exclusions at each stage, ideally using a flow diagram. | Yes; Page 10, Figure 1 |
| Characteristics of sources of evidence | 15 | For each source of evidence, present characteristics for which data were charted and provide the citations. | Yes; Appendix 2 |
| Critical appraisal within sources of evidence | 16 | If done, present data on critical appraisal of included sources of evidence (see item 12). | NA |
| Results of individual sources of evidence | 17 | For each included source of evidence, present the relevant data that were charted that relate to the review questions and objectives. | Yes; Page 10-12 |
| Synthesis of results | 18 | Summarize and/or present the charting results as they relate to the review questions and objectives. | Yes; Page 10-27 |
| **DISCUSSION** | | | |
| Summary of evidence | 19 | Summarize the main results (including an overview of concepts, themes, and types of evidence available), link to the review questions and objectives, and consider the relevance to key groups. | Yes; Page 27-33 |
| Limitations | 20 | Discuss the limitations of the scoping review process. | Yes; Page 35 |
| Conclusions | 21 | Provide a general interpretation of the results with respect to the review questions and objectives, as well as potential implications and/or next steps. | Yes; Page 35-36 |
| **FUNDING** | | | |
| Funding | 22 | Describe sources of funding for the included sources of evidence, as well as sources of funding for the scoping review. Describe the role of the funders of the scoping review. | Yes; Page 10, Appendix 2, Title page |

JBI = Joanna Briggs Institute; PRISMA-ScR = Preferred Reporting Items for Systematic reviews and Meta-Analyses extension for Scoping Reviews.

* Where *sources of evidence* (see second footnote) are compiled from, such as bibliographic databases, social media platforms, and Web sites.

† A more inclusive/heterogeneous term used to account for the different types of evidence or data sources (e.g., quantitative and/or qualitative research, expert opinion, and policy documents) that may be eligible in a scoping review as opposed to only studies. This is not to be confused with *information sources* (see first footnote).

‡ The frameworks by Arksey and O’Malley (6) and Levac and colleagues (7) and the JBI guidance (4, 5) refer to the process of data extraction in a scoping review as data charting*.*

§ The process of systematically examining research evidence to assess its validity, results, and relevance before using it to inform a decision. This term is used for items 12 and 19 instead of "risk of bias" (which is more applicable to systematic reviews of interventions) to include and acknowledge the various sources of evidence that may be used in a scoping review (e.g., quantitative and/or qualitative research, expert opinion, and policy document).

*From:* Tricco AC, Lillie E, Zarin W, O'Brien KK, Colquhoun H, Levac D, et al. PRISMA Extension for Scoping Reviews (PRISMAScR): Checklist and Explanation. Ann Intern Med. 2018;169:467–473. [doi: 10.7326/M18-0850](http://annals.org/aim/fullarticle/2700389/prisma-extension-scoping-reviews-prisma-scr-checklist-explanation).
